# P01-028 – MEFV mutation in Morrocan child wuth familial Mediterranean fever

**DOI:** 10.1186/1546-0096-11-S1-A32

**Published:** 2013-11-08

**Authors:** B Chkirate, A Dibi, A Bentahila

**Affiliations:** 1 Faculte de medecine, Rabat, Morocco

## Introduction

Familial Mediterranean Fever ( FMF ) is an autosomal recessive inherited disease mostly wide spread in the Mediterranean basin. It is manifested by a fever associated with paroxystic painful attacks. The prognosis is determined by the occurrence of renal amyloidosis. The purpose of our work is to establish a genotype- phenotype correlation between the MEFV gene mutation and the expression of the FMF in 10 Moroccan children.

## Case report

### Material and methods

It’s a retrospective study of children responding to the FMF Yalcinkaya criteria screened at infantile hospital of Rabat. The genetic study was conducted at the hygienic national institute of Rabat.

### Results

There are 6 boys and 4 girls at the average of 10 years old. The consanguinity was found in 2 cases. Similar familial cases were found in 3 cases. Fever and abdominal pain were present in all cases. Articular pains in 60% of cases and muscular ones in 30%. An inflammatory syndrome was found in all cases. The renal tests were normal in all cases. The genetic study revealed the presence of the MEFV gene mutation in 5 cases (50%): M694I in 2 cases, M694V in 1 case, M694V/M694I at a composite state in 1 case and M680I in 1 case. All the patients received colchicine. The evolution was favorable in 9 cases. The biotherapy was done to one patient because of the persistence of clinical symptomatology.

## Discussion

The “pathogene” effect of MEFV gene mutations is too variable. The foundering mutations M694V, M694I and M680I , which are very frequent in the populations at risk of FMF are also those linked to the most severe phenotypes. Nevertheless, variable penetrance and expression of FMF could be explained by the type and number of mutation but also by other modifiers genes and/or environmental factors.

## Disclosure of interest

None declared.

